# A Randomized Controlled Trial of Chinese Medicine on Nonmotor Symptoms in Parkinson's Disease

**DOI:** 10.1155/2017/1902708

**Published:** 2017-05-23

**Authors:** Ka-Kit Chua, Adrian Wong, Kam-Wa Chan, Yin-Kei Lau, Zhao-Xiang Bian, Jia-Hong Lu, Liang-Feng Liu, Lei-Lei Chen, Ka-Ho Chan, Kim-Pong Tse, Anne Chan, Ju-Xian Song, Justin Wu, Li-Xing Zhu, Vincent Mok, Min Li

**Affiliations:** ^1^School of Chinese Medicine, Hong Kong Baptist University, Kowloon, Hong Kong; ^2^Mr. & Mrs. Ko Chi Ming Centre for Parkinson's Disease Research, Hong Kong Baptist University, Kowloon, Hong Kong; ^3^Institutes of Integrative Medicine, Department of Medicine and Therapeutics, The Chinese University of Hong Kong, Sha Tin, Hong Kong; ^4^State Key Laboratory of Quality Research in Chinese Medicine, Institute of Chinese Medical Sciences, University of Macau, Macau; ^5^Department of Mathematics, Statistics Research & Consultancy Centre, Faculty of Science, Hong Kong Baptist University, Kowloon, Hong Kong

## Abstract

Nonmotor symptoms (NMS) of Parkinson's disease (PD) have devastating impacts on both patients and their caregivers. Jiawei-Liujunzi Tang (JLT) has been used to treat some NMS of PD based on the Chinese medicine theory since Qing dynasty. Here we report a double-blind, randomized, placebo-controlled, add-on clinical trial aiming at evaluating the efficacy and safety of the JLT in treating NMS in PD patients. We randomly assigned 111 patients with idiopathic PD to receive either JLT or placebo for 32 weeks. Outcome measures were baseline to week 32 changes in Movement Disorder Society-Sponsored Revision of Unified PD Rating Scale (MDS-UPDRS) Parts I–IV and in NMS assessment scale for PD (NMSS). We observed improvements in the NMSS total score (*p* = 0.019), mood/cognition (*p* = 0.005), and reduction in hallucinations (*p* = 0.024). In addition, post hoc analysis showed a significant reduction in constipation (*p* < 0.001). However, there was no evidence of improvement in MDS-UPDRS Part I total score (*p* = 0.216) at week 32. Adverse events (AEs) were mild and comparable between the two groups. In conclusion, long-term administration of JLT is well tolerated and shows significant benefits in improving NMS including mood, cognition, and constipation.

## 1. Introduction

Parkinson's disease (PD) is the second most common neurodegenerative disease in the world, with a prevalence rate of 1% in the population over age of 60 [1]. Increasing attention has been paid to nonmotor aspects which might precede motor symptoms [2]. Common nonmotor symptoms (NMS) of PD include fatigue, mood disorders, hallucinations, constipation, and sleep disorders [3]. Though not fatal, they reduce quality of life for both patients and their caregivers [4]. The most common treatment for PD is levodopa. However, levodopa primarily treats motor symptoms and it typically generates adverse events after long-term use [5]. As a result of both the failure of levodopa improving NMS and its side effects, patients often seek alternative treatments [6].

Traditional Chinese medicine (TCM) is one of the most investigated streams of alternative medicine [7, 8]. It has been used to treat PD throughout China [9]. In TCM theory, patients are divided into categories according to the signs and symptoms presented [10]. The concept is similar to using factor analysis and cluster analysis in modern statistics to classify patients with different clinical patterns [11]. According to Chinese medicine theory, PD patients who present with fatigue, constipation, and/or mood disorder are classified in the subgroup of “Spleen Qi Deficiency.” Treatment typically involves different herbal formulas to “Replenish Spleen Qi.” Randomized controlled trials (RCT) have been conducted to examine the efficacy and safety of using various TCM formulas to treat PD. However, the quality of most of these RCT is compromised by methodological defects including poor randomization, insufficient masking, lack of proper sample size calculation, and/or improper data analysis [12].

Our group previously reported that a Chinese herbal medicine formula, Jiawei-Liujunzi Tang (JLT), relieved some nonmotor complications after 24 weeks of treatment [13]. It has been used to treat some PD-like NMS since Qing dynasty [13]. Recently, our team demonstrated that corynoxine B (Cory B), an active compound isolated from the Chinese medicine* Uncaria rhynchophylla *(Miq.) Jacks. (Gouteng in Chinese), which is one of the principal herbs in the JLT, efficiently promotes the clearance of *α*-synuclein (*α*-syn) aggresomes in vitro and in vivo via inducing autophagy which protects neurons in PD [14]. Cory B rescues *α*-syn-induced impairment of autophagy, possibly through blocking *α*-syn-HMGB1 (high mobility group box 1, HMGB 1) interaction [15]. Moreover, our group investigated corynoxine (Cory), another active compound isolated from Gouteng, and found that it can promote the clearance of *α*-syn via Akt/mTOR (Akt murine thymoma viral oncogene homolog 1, Akt; mammalian target of rapamycin, mTOR) pathway [16]. We also found that more than 90% of PD patients are having DSQ [17]. JLT may be suitable for most PD patients if it is workable.

Given the number of people using Chinese medicine, it is critical to test the efficacy of JLT in clinical trials. In this trial, we aimed to study the efficacy and safety of using JLT to treat NMS in idiopathic PD patients.

## 2. Methods

### 2.1. Participants

*Inclusion Criteria*. Adults between 18 and 80 years of age who (1) had been diagnosed with idiopathic PD based on UK Brain Bank criteria with Hoehn and Yahr (H&Y) stages 1–4 by conventional medicine physicians [18] and (2) presented symptoms classified as Deficiency of Spleen Qi (diagnosis of DSQ, Supplementary Material 1, available online at https://doi.org/10.1155/2017/1902708) based on Guidance for Clinical Research of New Chinese Herbal Medicine published by China [19] during a screening visit were eligible. The diagnostic criteria of DSQ included presentation of dyspepsia, fatigue, and abdominal distention. Other inclusion criteria included stable daily administration of levodopa and permitted antiparkinsonian drugs (dopamine agonists, selegiline, rasagiline, entacapone, amantadine, and anticholinergic drugs) for at least 4 weeks before the start of treatment and normal liver and renal function.

*Exclusion Criteria*. Patients who had atypical or drug-induced parkinsonism, a score of <24 on the Mini-Mental State Examination (MMSE), history of psychosis, history of Chinese herbal medicine allergy, concurrent intake of antidepressants, a history of suicide attempts, or unstable medical disorders were excluded. Those who had participated in other trials within 30 days of the start of this trial as well as women who were pregnant or were breastfeeding were also excluded.

This clinical study was carried out at the Hong Kong Baptist University (HKBU) Chinese Medicine Specialty Centre. It was approved by the Ethics Committee of the HKBU's Institutional Review Board (code: HASC/09-10/09) and registered on the Chinese Clinical Trial Registry (ChiCTR-TRC-13003085). Written informed consent was obtained from every patient before they participated in any study-related activity. This study report followed the guidelines of Consolidated Standards of Reporting Trials (CONSORT).

### 2.2. Sample Size Calculation

According to our previous pilot study [13], the management team estimated an effect size of 0.626 and a standard deviation of 1.99 with G-Power version 3.1. At least 105 patients (1 : 1) were required to provide an 80% power of detecting a difference with a 2-sided*α*-level of 0.05 with a maximum of 20% attrition rate. No covariates or center effects were used in power calculation.

### 2.3. Randomization and Masking

This study was a double-blind, randomized, placebo-controlled, add-on trial. Patients were randomly assigned to receive 32 weeks of either active herbal treatment or placebo and followed up for a further 6-week observation period without treatment. The randomization sequence was generated by “Random Allocation Software.” The sequence was password-protected and kept in a computer by Lei-Lei Chen. Group allocation was stratified block randomization according to their H&Y stages at the screening. The sequential number was contained in a sealed opaque envelop and distributed to assessors. Patients, investigators, and all sponsoring parties were masked to treatment allocation until the end of the study.

### 2.4. Study Medication

The active herbal medicine under study was JLT (Supplementary Table  1, composition of JLT). The granules were produced in a single batch (JLT batch number: A120065; Placebo batch number: A120153), mixed, and packed to ensure the stability and homogeneity of the composition by PuraPharm Pharmaceuticals Company Limited, a GMP plant, as previously reported [13]. The placebo was made of caramel, gardenia yellow pigment, sunset yellow, permicol egg yellow, cocoa brown, citric acid, sodium cyclamate, dextrin, and broadleaf holly leaf [20]. The herbal granules and the placebo granules had identical appearance and smell, and both were sealed in plastic bags. All herbal and placebo granules were distributed by Kim-Pong Tse with both written and verbal instructions for each participant. They were instructed to take the granules orally, twice per day, 11 g each time (a dosage equivalent to 55 g herbs), at least two hours apart from taking any routine Western medication.

### 2.5. Outcome Measurements and Its Assessment

The primary outcome of this study was the Movement Disorder Society-Sponsored Revision of Unified PD Rating Scale (MDS-UPDRS) [21] Part I total score. MDS-UPDRS Part I subscores, nonmotor symptom assessment scale (NMSS) for Parkinson's disease [22] total score, and total scores of each domain as well as the total score of other parts of MDS-UPDRS (Parts II–IV) were used as secondary outcomes.

Outcome measurements were carried out during the study visits at weeks 0, 16, 32 (end of treatment), and 38 (end of observation period). Assessments were carried out in the “on” state. Safety assessment, which included reporting of adverse events (AEs) and measurement of vital signs and physical examination, was carried out throughout the study. In addition, laboratory safety screening of liver and renal function was performed at week 32. Both bilingual assessors, that is, Ka-Kit Chua and Yin-Kei Lau, were blind to the allocation and were trained by the same neurology specialist Vincent Mok and qualified by the online training program of the Movement Disorder Society.

A home diary was given to the patients or their caregivers to monitor their medical condition. Formal instruction for the home diary was given during the first visit. Compliance to treatment was defined by the record of the diary with reference to the amount of the returned medicine/placebo packages.

### 2.6. Statistical Analysis

Demographic and clinical data were compared between the JLT and placebo groups using independent sample* t*-test or Chi-squared test as appropriate. Changes in primary and secondary outcomes, between baseline and week 32, were compared between the JLT and placebo groups using independent sample* t*-tests.

Missing data were input in the last-observation-carried-forward (LOCF) manner. All patients randomized with at least one postrandomization measurement were included in the primary analysis to follow the intention-to-treat principle. Analyses were done with SPSS 19.0 package (SPSS, Chicago, IL).

To avoid inflation of type-1 errors due to multiple-endpoint testing, analyses of the primary outcomes were performed with a hierarchical approach. To begin, the scores of MDS-UPDRS Part I at week 32 for the JLT and placebo groups were compared. If the difference was deemed statistically significant at a 2-sided*α*-level of 0.05, the scores of MDS-UPDRS Part I at week 16 and week 38 were compared between groups. The hierarchical order was as follows: (1) MDS-UPDRS Part I total score at week 32; (2) MDS-UPDRS Part I total score at weeks 16 and 38. Secondary outcomes were analyzed in the same manner as the primary outcome.

A post hoc analysis was performed to test any possible effect of JLT by analyzing all the subscores of each domain of NMSS in the same manner as the primary outcome.

## 3. Results

**Figure 1:**
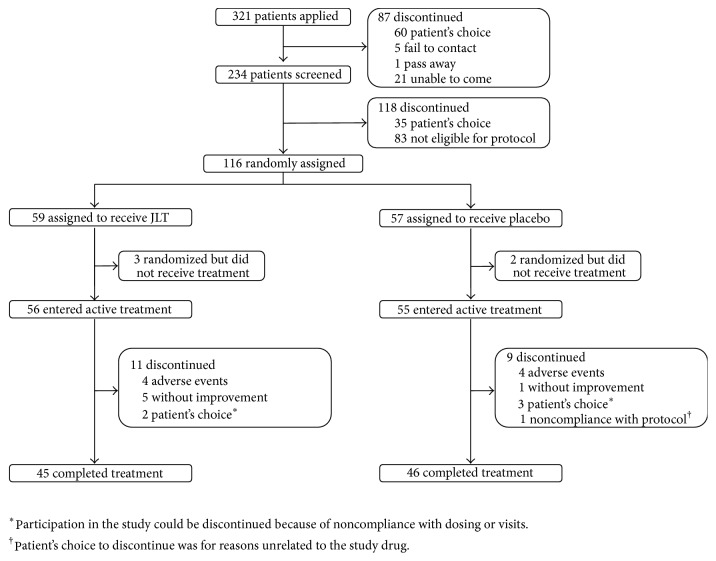


Figure 1 is a flow chart depicting the participant screening and recruitment in this study. Demographic data and baseline scores are summarized in Table 1. A total of 234 patients were screened for eligibility, and 116 participants were enrolled. Five patients withdrew from the study due to personal reasons after randomization and before the start of treatment. Among the remaining 111 patients (73 males; 38 females; mean ages: 62.69  ±  9.11 years; mean duration of PD: 5.95  ±  3.97 years), 56 were assigned to the JLT group and 55 were assigned to the placebo group. Twenty participants dropped out during the study due to reasons listed in Figure 1. Forty-five participants in the JLT group and 46 in the placebo group completed the study.

In the primary analysis, we observed a trend of improvement; a decreased score was obtained at week 32 in JLT group which suggested an improvement in NMS in the MDS-UPDRS Part I total score in the JLT group relative to the placebo group, though the difference was not statistically significant (mean diff. = −1.30; 95% CI: −3.37 to 0.77; *p* = 0.215). In comparison, an increased score was obtained at week 32 in the placebo group, which suggested worsening. Further analyses performed on the subscores of MDS-UPDRS Part I between the two groups revealed that the JLT group showed nonsignificant trends of reduction in constipation (mean diff. = −1.09; 95% CI: −2.30 to 0.13; *p* = 0.079) and in hallucination (mean diff. = −0.18; 95% CI: −0.38 to 0.19; *p* = 0.075) compared to the placebo group (Table 2).

In the secondary analysis, the data indicate a significant difference in NMSS for Parkinson's disease total score between JLT group and placebo group (mean diff. = −14.05; 95% CI: −25.71 to −2.39; *p* = 0.019) after 32 weeks of treatment. A trend of improvement in the JLT group was also noted by the hierarchical approach in week 16 (mean diff. = −8.87; 95% CI: −18.78 to 1.04; *p* = 0.079) and the improvement persisted at 38 weeks (mean diff. = −11.70; 95% CI: −23.12 to −0.28; *p* = 0.045). Further analyses of the NMSS subscores showed that the PD patients in the JLT group experienced improvement in mood/cognition (mean diff. = −6.66; 95% CI: −11.24 to −2.09; *p* = 0.005) and reduction in hallucinations (mean diff. = −1.58; 95% CI: −2.96 to −0.21; *p* = 0.024) compared to those in the placebo group at week 32. Relative to the control group, the JLT group showed a trend of improvement in the gastrointestinal tract (mean diff. = −1.84; 95% CI: −3.87 to −0.20; *p* = 0.076) as well as a significant reduction in constipation which persisted from week 16 (mean diff. = −2.49; 95% CI: −3.75 to −1.23; *p* < 0.001) to week 38 (mean diff. = −2.17; 95% CI: −3.53 to −0.82; *p* = 0.002) (Table 3). There were no significant differences in other subscores between the two groups at week 16 and the end of treatment in other domains. No statistically significant differences were found in other parts (II–IV) of MDS-UPDRS in week 32.

For the withdrawal and adverse events, twenty patients (11 (19.6%) [JLT] versus 9 (16.4%) [placebo], *p* = 0.65) discontinued treatment after randomization. Among these 20 patients, 4 in each group withdrew because of AEs. During the treatment phase, two patients (3.57%) in the JLT group and four patients (7.27%) in the placebo group had serious AEs: one patient had hypoglycemia (placebo), one had sepsis (placebo), one had finger sarcoma (placebo), two had coronary heart disease (one in placebo and one in JLT), and one had breast cancer (JLT). No deaths were recorded during the trial. AEs were reported by at least 5% of patients in each group; these are presented in Table 4.

## 4. Discussion

In this RCT, there was no evidence supporting the hypothesis that JLT can reduce NMS as represented by improvement in the overall MDS-UPDRS Part I score (the NMS of PD). However, a reduction in NMS was noted by the secondary outcome of the NMSS total score, even after the patients had stopped medication. Also, improvement in the form of reduction in hallucinations and constipation was suggested by secondary analysis of MDS-UPDRS Part I subscore. Improvement in the mood, hallucinations, and constipation, without effect on the motor features of PD, was found by secondary analysis and post hoc analysis of the NMSS subscore. JLT was well tolerated. Discontinuation due to AEs occurred with the same frequency in the JLT group (4 patients) as in the placebo group (4 patients). Further targeted studies on the effect of JLT on mood and gastrointestinal condition could confirm these observations.

TCM has long been used to treat symptoms similar to PD in China [23]. According to TCM theory, JLT replenishes and facilitates circulation of “spleen and stomach Qi,” which is related to PD NMS. Depletion and stagnation of “spleen and stomach Qi” would lead to NMS such as constipation, nausea, sleep disruption, and mood disorder. In this trial, the improvement of NMS assessed by the NMSS showed continual effect even after the patients had stopped the medication for 6 weeks. It suggested that the effect of JLT may not be just symptomatic; instead, it may alter some pathophysiological processes underlying NMS.

JLT is an herbal formula composed mainly of* Codonopsis pilosula *(Franch.) Nannf. (Dangshen in Chinese),* Rehmannia glutinosa *Libosch. (Dihuang in Chinese),* Poria cocos *(Schw.) Wolf. (Fuling in Chinese), and* Uncaria rhynchophylla* (Miq.) Jacks. (Gouteng in Chinese) [13]. Jung et al. found that Gouteng extract is an effective anxiolytic agent and acts via the serotonergic nervous system [24]. Lee et al. showed that triterpenoids in Fuling may regulate the expressed 5-hydroxytryptamine 3A (5-HT_3A_) receptors which have close relationship to the gastric system and nervous system [25]. These suggest that the effect of JLT, which includes improvement in mood and reduction of constipation, may be due to the increased levels or stimulation of serotonin receptors.

Serotonin or 5-hydroxytryptamine (5-HT) is a monoamine neurotransmitter with a significant role in mood and appetite regulation [26]. It is synthesized in both serotonergic neurons of the central nervous system (CNS) to regulate mood and appetite and in the alimentary canal to regulate intestinal movements [27]. Neurotransmitters in general, 5-HT in particular, may be involved in the NMS of PD, including mood disorder, psychosis, and constipation [28].

For the limitations, the improvement of NMS was just supported by the NMSS total score (*p* = 0.019), the secondary outcome, but not the MDS-UPDRS Part I total score (*p* = 0.216), the primary outcome. Also, constipation was improved by JLT as suggested by the post hoc analyses of NMSS. We are aware that the evidence suggesting improvements in constipation (*p* = 0.079) by the MDS-UPDRS Part I, the secondary outcome, among patients taking JLT was noted to be weak. The inconsistency within the test may be due to the inadequate power of the study to measure small differences. It should be noted though that while the differences are small, to an individual patient, this small difference may still have an important impact on their quality of life. The current data suggests that a scaled-up study would be able to confirm the difference [29]. On the other hand, the scale of NMSS is more in depth in testing NMS than the MDS-UPDRS (score: 0–4). NMSS measures the severity as well as the frequency of each NMS independently and multiplies these factors to achieve an overall result (score: 0–12). This may result in a difference between the two measurements.

An attrition rate of 20% was high for PD patients at a relatively early stage of the disease. This was due to the long treatment period (32 weeks) when compared to other clinical trials [30]. As the main motivation of some PD patients to participate in a clinical trial was to obtain benefit [31], 6 patients withdrew when they believed the medication was ineffective.

Another limitation is that there was a significant difference at baseline in the number of patients using catechol O-methyltransferase (COMT) inhibitor and the total score of NMSS and MDS-UPDRS Part II even after randomization. This might be due to the use of stratified block randomization based on the H&Y stages of PD patients. PD patients were divided into difference H&Y stages based on their clinical motor symptoms, which was best shown by the total score of MDS-UPDRS Part 3. As H&Y stages do not consider the use of medication, this may result in differences in the medical history of patients. In general, the more medicines patients are taking, the more serious their condition becomes. It could induce a great variation in the assessment score even if the patients were in the same PD stage. Hence, a difference could result in the motor part and nonmotor part but not in the clinical presentation of PD symptoms, the total score of MDS-UPDRS Part 3. Larger sample size may be a possible way to minimize such problem in future.

In conclusion, although the result of MDS-UPDRS does not show significant improvement, the NMSS data does show some positive outcomes on the NMS of PD. Our data suggest that JLT could alleviate gastrointestinal problems and mood disorders in some PD patients over 32 weeks with minimal side effects. Also, the effect of JLT on NMS and constipation could persist 6 weeks after treatment. It appears to be both safe and effective for long-term use to treat NMS of PD. While not conclusive, this initial trial warrants future work into JLT, especially on the mood and gastrointestinal improvement of PD patients.

## Supplementary Material

Supplementary Material 1: Diagnostic criteria for deficiency of spleen qi (DSQ). Supplementary Table 1: JLT composition.

## Figures and Tables

**Table 1:** Baseline characteristics.

Parameter	JLT group (*n* = 56)	Control group (*n* = 55)	*p* value^a^
Age (years)	63.48 ± 9.72	63.31 ± 8.20	0.919^b^
Gender (M/F)	35/21	38/17	0.464^c^
Disease duration (years)	6.42 ± 4.15	5.42 ± 3.77	0.096^b^
Duration of Levodopa treatment (years)	5.17 ± 4.42	3.94 ± 3.13	0.187^b^
Total Levodopa dosage (mg/day)	459.82 ± 350.90	374.55 ± 257.46	0.148^b^
Medication use			
Levodopa, *n* (%)	53 (94.6)	50 (90.9)	0.447^c^
Dopaminergic agonist, *n* (%)	23 (41.1)	17 (30.9)	0.265^c^
Anticholinergic, *n* (%)	18 (32.1)	21 (38.2)	0.505^c^
COMT inhibitor, *n* (%)	14 (25.0)	4 (7.3)	0.011^c^
MAO-B inhibitor, *n* (%)	17 (30.4)	12 (21.8)	0.306^c^
Amantadine, *n* (%)	5 (8.9)	4 (7.3)	0.749^c^
Senna, *n* (%)	5 (8.9)	6 (10.9)	0.727^c^
Lactulose, *n* (%)	4 (7.1)	3 (5.5)	0.714^c^
Baseline scores			
H&Y score	2.07 ± 0.60	2.02 ± 0.59	0.639^b^
NMSS total	65.52 ± 49.77	47.42 ± 35.70	0.030^b^
MDS-UPDRS part I	10.21 ± 7.06	8.76 ± 6.63	0.267^b^
MDS-UPDRS part II	14.71 ± 7.95	11.58 ± 7.51	0.035^b^
MDS-UPDRS part III	33.21 ± 15.39	33.27 ± 14.27	0.983^b^
MDS-UPDRS part IV	4.00 ± 4.45	2.78 ± 3.70	0.120^b^

Data are expressed as mean ± S.D; ^a^*p* value was comparing the difference between two groups in baseline; ^b^treatment group compared with placebo group by independent *t*-test; ^c^treatment group compared with placebo group by Chi-square test with continuity correction.

**Table 2:** Efficacy result of JLT on Parkinson's disease patient at week 32.

	JLT	Placebo	Mean difference	95% confidence interval (CI)	*p* value^a^
Movement Disorder Society-Sponsored Revision of Unified PD Rating Scale					
(MDS-UPDRS)					
Part I total score (nonmotor symptom)	−0.46 ± 6.61	0.84 ± 4.09	−1.30	−3.37 to 0.77	0.215
Q.1 Cognitive	−0.07 ± 0.97	0.00 ± 0.69	−0.07	−0.39 to 0.25	0.657
Q.2 Hallucinations	−0.04 ± 0.54	0.15 ± 0.52	−0.18	−0.38 to 0.02	0.075
Q.3 Depression	−0.07 ± 1.03	0.07 ± 0.72	−0.14	−0.48 to 0.19	0.393
Q.4 Anxious	−0.14 ± 0.82	−0.02 ± 0.71	−0.13	−0.41 to 0.16	0.393
Q.5 Apathy	−0.23 ± 1.36	−0.02 ± 0.87	−0.21	−0.64 to 0.22	0.327
Q.6 Dopamine dysregulation	−0.13 ± 0.63	−0.15 ± 0.52	0.02	−0.20 to 0.24	0.854
Q.7 Sleep	0.23 ± 1.03	0.13 ± 1.06	0.11	−0.29 to 0.50	0.597
Q.8 Daytime sleep	0.16 ± 0.91	0.11 ± 0.85	0.05	−0.28 to 0.38	0.759
Q.9 Pain	−0.02 ± 1.30	0.24 ± 1.05	−0.25	−0.70 to 0.19	0.261
Q.10 Urinary	−0.02 ± 0.73	0.02 ± 0.87	−0.04	−0.34 to 0.27	0.813
Q.11 Constipation	−0.13 ± 1.45	0.96 ± 4.35	−1.19	−2.30 to 0.13	0.079
Q12 Light headedness	0.04 ± 1.08	−0.04 ± 0.38	0.72	−0.23 to 0.38	0.641
Q13 Fatigue	−0.04 ± 1.21	−0.02 ± 0.97	−0.05	−0.47 to 0.36	0.796
Part II total score (motor symptom)	0.45 ± 4.13	1.05 ± 4.58	−0.61	−2.25 to 1.03	0.464
Part III total score (motor examination)	−0.52 ± 10.13	1.38 ± 8.27	−1.90	−5.38 to 1.58	0.282
Part IV total score (motor complications)	0.21 ± 3.56	0.65 ± 3.42	−0.44	−1.75 to 0.87	0.508

Nonmotor Symptom Assessment Scale for Parkinson's Disease					
(NMSS)					
Total score	−2.27 ± 32.90	11.78 ± 28.93	−14.05	−25.71 to −2.39	0.019
D1 total cardiovascular	−0.89 ± 3.38	−0.15 ± 2.31	−0.75	−1.84 to 0.35	0.178
D2 total sleep/fatigue	3.63 ± 7.98	4.62 ± 7.08	−0.99	−3.83 to 1.84	0.490
D3 total mood/cognition	−3.54 ± 14.38	3.13 ± 9.36	−6.66	−11.23 to −2.10	0.005
D4 total perceptual/hallucinations	−0.88 ± 4.17	0.71 ± 3.04	−1.58	−2.96 to −0.21	0.024
D5 total attention/memory	−1.86 ± 6.07	−0.15 ± 7.08	−1.71	−4.20 to 0.77	0.174
D6 total gastrointestinal tract	−0.86 ± 5.15	0.98 ± 5.66	−1.84	−3.87 to 0.20	0.076
D7 total urinary	1.71 ± 7.57	1.47 ± 7.12	0.24	−2.52 to 3.01	0.863
D8 total sexual function	−0.71 ± 4.20	0.16 ± 3.81	−0.88	−2.39 to 0.63	0.251
D9 total miscellaneous	1.13 ± 6.41	1.00 ± 5.46	0.13	−2.11 to 2.36	0.912

^a^ *p* value wascomparing the score changes at week 32 between JLT group and placebo group by independent sample *t*-tests; values are given as mean ± S.D. Values in JLT group and placebo group are the score changed in the same group between week 32 and baseline (score at week 32 minus score at the baseline).

**Table 3:** Result of hierarchical approach.

Parameter	Week 16		Week 32		Week 38	
	JLT	Placebo	JLT	Placebo	JLT	Placebo
Total score of NMSS	−3.13 ± 29.49	5.75 ± 22.67	−2.27 ± 32.90	11.78 ± 28.93	0.48 ± 34.01	12.18 ± 26.08
	Mean difference: −8.87		Mean difference: −14.05		Mean difference: −11.70	
	95% CI: −18.78 to 1.04		95% CI: −25.71 to −2.39		95% CI: −23.12 to −0.28	
	*p* value = 0.079		*p* value = 0.019		*p* value = 0.045	

NMSS D3 total mood/cognition	−1.68 ± 12.12	0.93 ± 7.76	−3.54 ± 14.38	3.13 ± 9.36	−0.66 ± 15.02	3.18 ± 10.38
	Mean difference: −2.61		Mean difference: −6.66		Mean difference: −3.84	
	95% CI: −6.43 to 1.22		95% CI: −11.23 to −2.10		95% CI: −8.71 to 1.02	
	*p* value = 0.181		*p* value = 0.005		*p* value = 0.120	

NMSS D4 total perceptual/hallucinations	−0.64 ± 3.28	0.09 ± 0.59	−0.88 ± 4.17	0.71 ± 3.04	−0.86 ± 4.52	0.22 ± 1.76
	Mean difference: −0.73		Mean difference: −1.58		Mean difference: −1.08	
	95% CI: −1.62 to 0.16		95% CI: −2.96 to −0.21		95% CI: −2.37 to 0.22	
	*p* value = 0.105		*p* value = 0.024		*p* value = 0.103	

NMSS D6 Q21 constipation	−1.02 ± 3.89	1.47 ± 2.71	−1.25 ± 3.46	1.55 ± 3.40	−0.43 ± 3.68	1.75 ± 3.54
	Mean difference: −2.49		Mean difference: −2.80		Mean difference: −2.17	
	95% CI: −3.75 to −1.23		95% CI: −4.09 to −1.50		95% CI: −3.53 to −0.82	
	*p* value < 0.001		*p* value < 0.001		*p* value = 0.002	

*p* value was comparing the score changes at different time points between JLT group and placebo group by independent sample *t*-tests; values are given as mean ± S.D. Values in JLT group and placebo group are the score changed in the same group between different time points and baseline (score at different time points minus score at the baseline).

**Table 4:** Adverse events reported by >5% of patients in each group.

Adverse events	Number of patients (%)	
	JLT (*N* = 56)	Placebo (*N* = 55)
Abdominal pain	3 (5.36)	3 (5.45)
Dyspepsia	5 (8.93)	1 (1.82)
Diarrhea	1 (1.79)	3 (5.45)
Dizziness	3 (5.36)	7 (12.73)
Back pain	1 (1.79)	5 (9.09)
Joint pain	2 (3.57)	4 (7.27)
