# Supplementary material for: A Randomized Controlled Trial of Chinese Medicine on Nonmotor Symptoms in Parkinson's Disease
Source: Parkinsons Dis. 2017 May 23;2017:1902708. doi: 10.1155/2017/1902708 (PMC5463168; doi:10.1155/2017/1902708)
Supplement: Supplementary file 1 — Supplementary Material 1: Diagnostic criteria for deficiency of spleen qi (DSQ). Supplementary Table 1: JLT composition. [file 1902708.f1.docx]

**Supplementary Material 1. Diagnostic criteria for deficiency of spleen qi (DSQ)**

**Diagnostic Criteria for Deficiency of Spleen Qi:**

(1) Primary symptoms: dyspepsia and anorexia; fatigue; abdominal distention after meal or in the afternoon; abnormal defecation (loose stool, alternate constipation and diarrhea, irregular constipation and diarrhea)

(2) Secondary symptoms: listless and reticent; dysgeusia; continuous abdominal pain; nausea and vomiting; gastric discomfort; gurgling sound; sallow complexion; edema; Inability defecation; pale tongue; thick tongue or with teeth mark; tongue with thin and white fur; thin and faint pulse.

For the diagnosis of deficiency of spleen Qi, patients must have at least 2 primary symptoms, or have 1 primary symptom plus 2 or more secondary symptoms.

Reference: Zheng XY. *The Guidance for Clinical Research of New Chinese Herbal Medicine*. Beijing, Chinese Medicine Scientific Publishers. 2002. p 361-364.

**Supplementary Table 1. JLT composition**

| **Chinese name** | **Pharmaceutical name** | **Percentage** |
| --- | --- | --- |
| Dang Shen | Dried root of *Codonopsis pilosula* (Franch.) Nannf. (Fam. Campanulaceae) | 13.39 |
| Sheng Di | Dried root tuber of *Rehmannia glutinosa* Libosch. (Fam. Scrophulariaceae) | 13.39 |
| Fu Ling | Dired sclerotium of the fungus, *Poria cocos* (Schw.) Wolf. (Fam. Polyporaceae) | 10.71 |
| Gou Teng | Dried hook-bearing stem branch of *Uncaria rhynchophylla* (Miq.) Jacks. (Fam. Rubiaceae) | 10.71 |
| Bai Zhu | Rhizome of *Atractylodes macrocephala* Koidz. (Fam. Compositae) | 8.93 |
| Dang Gui | Dried root of *Angelica sinensis* (Oliv) Diels. (Fam. Umbelliferae) | 8.93 |
| Fa Ban Xia | Dried tuber of *Pinelliae ternate* (Thunb.) Breit. (Fam. Araceae) | 8.04 |
| Chuan Xiong | Dried rhizome of *Ligusticum chuanxiong* Hort. (Fam. Umbelliferae) | 8.04 |
| Huai Niu Xi | Dried root of *Achyranthes bidentata* BI. (Fam. Amaranthaceae) | 8.04 |
| Chen Pi | Dried pericarp of the ripe fruit of *Citrus reticulate* Blanco. (Fam. Rutaceae) | 5.36 |
| Sheng Gan Cao | Dried root and rhizome of *Glycyrrhiza uralensis* Fisch. (Fam. Leguminosae) | 4.46 |
